# The Association Between Medication Use in Older Women with Early-Stage Operable Primary Breast Cancer and Decision Regarding Primary Treatment

**DOI:** 10.1093/oncolo/oyac278

**Published:** 2023-01-30

**Authors:** Natalie Tse, Ruth M Parks, Holly M Holmes, Kwok-Leung Cheung

**Affiliations:** University Hospitals of Derby and Burton NHS Foundation Trust, Derby, UK; Nottingham Breast Cancer Research Centre, University of Nottingham, Nottingham, UK; Nottingham Breast Cancer Research Centre, University of Nottingham, Nottingham, UK; School of Medicine, University of Nottingham, Nottingham, UK; Division of Geriatric and Palliative Medicine, University of Texas Health Science Center, McGovern Medical School, Houston, TX, USA; Nottingham Breast Cancer Research Centre, University of Nottingham, Nottingham, UK; School of Medicine, University of Nottingham, Nottingham, UK

**Keywords:** polypharmacy, breast cancer, medications, functional status, treatment, older women

## Abstract

**Background:**

Polypharmacy is one factor contributing to increased mortality, hospitalization, and adverse drug reactions in older adults. The aim of this study was to measure the prevalence of polypharmacy in a cohort of older women with early-stage operable primary breast cancer and the relationship of polypharmacy to primary treatment decision and functional status.

**Methods:**

A total of 139 patients with a new diagnosis of early-stage operable primary breast cancer proven histologically were recruited as part of a prospective study. The average age was 77 years. Assessment using a cancer-specific Comprehensive Geriatric Assessment (CGA) tool was conducted within 6 weeks of diagnosis of breast cancer. Association was determined between number of medications and treatment decision and physical status as measured by the CGA outcomes. Additional analysis was performed to determine the associations above with polypharmacy defined by ≥5 daily medications, and if cardiovascular-related diseases have a role in the treatment decision.

**Results:**

Polypharmacy was present in 48% of patients (*n* = 139). CGA determined that polypharmacy was associated with greater comorbidity (*P* < .001), reduced physical status rated by physicians (*P* = .009) and patients (*P* = .019), and reduced ability to perform activities of instrumental ADLs (*P* = .008). Similar findings were present in the analysis of cardiovascular-related diseases.

**Conclusions:**

This work suggests that patients with polypharmacy are more likely to be frail. The number of medications could help us screen patients who should go on to receive full CGA.

## Teaser

This study measured the prevalence of polypharmacy in a cohort of older women with early-stage operable primary breast cancer and the relationship of polypharmacy to primary treatment decision and functional status.

**Implications for Practice**

Results of this study show a benefit to having a clinical pharmacist review medications for older women when they are first diagnosed with primary breast cancer. The screening process could involve assessment of the appropriateness of polypharmacy, de-prescribing of medications, organ function assessment, and drug interaction check. It may also be a useful tool to decide which patients should go on to receive a comprehensive geriatric assessment.

## Introduction

As the population is aging globally, the care of older patients has become increasingly challenging for healthcare systems. Cancer care in older people is no exception due to the high prevalence of cancers and the complexity of treatment in this population.^1,2^ Older patients often present with geriatric syndromes such as falls, delirium, cognitive impairment, depression, and polypharmacy. In oncology, the presence of one or more of these syndromes may affect treatment tolerance and clinical outcomes.^3^ Polypharmacy is particularly important as it is closely related to the incidence of these geriatric syndromes.^4^

### Definition of Polypharmacy

Polypharmacy is described as the use or prescribing of multiple medications to a person; however, to date, there is no international consensus on a unified numeric definition of polypharmacy.^1,5^ Depending on the setting and application, polypharmacy can be conceptualized as the number of regular medications or the appropriateness of prescribing as a qualitative approach.^5,6^ In a research setting, the use of a specific numeric threshold allows the replicability of studies.^5,7^ A cutoff of 5 or more medicines per day to define polypharmacy and 10 or more daily medications to describe hyperpolypharmacy are the definitions most frequently used in the literature.^5,7,8^

### Polypharmacy Association with Adverse Outcomes

In practice, polypharmacy often implies negative connotations of prescribing as it is recognized as a contributing factor to increased mortality, hospitalization, and adverse drug reactions (ADR) in older adults.^9–11^ Polypharmacy is associated with greater prescribing errors,^12,13^ higher risk of drug–drug interaction,^11^ and hospitalization.^14^ It is an important challenge in older patients with cancer as they navigate treatment decision-making alongside multiple comorbidities and frailty.^15,16^

Nevertheless, it should be borne in mind that polypharmacy can be entirely appropriate where evidence-based medicines are used to achieve the goals of care for patients.

### Comprehensive Geriatric Assessment in an Oncology Setting

Comprehensive geriatric assessment (CGA) has been widely researched and adapted in the care of older people. It is an interdisciplinary process involving not only clinical assessment but also evaluation of patient’s functional and mental status, social circumstances, and environment.^17^ Implementation of CGA is recommended by the International Society of Geriatric Oncology (SIOG) in order to achieve better clinical outcomes in older cancer patients.^18–20^ However, the major barriers to the implementation of CGA in clinical practice include length of time to complete the assessment, associated costs,^17^ and how to action findings.

CGA may be particularly useful to help guide treatment decision-making in older patients for whom alternative treatment approaches are offered based on comorbidities and likely life expectancy. Older women with breast cancer may particularly benefit from CGA to guide decision-making. According to the latest recommendations from the SIOG and the European Society of Breast Cancer Specialists, surgery remains the choice of primary treatment in older patients with early breast cancer. Primary endocrine therapy (PET) is considered an alternative treatment in patients with ER-positive breast cancer, or those with limited life expectancy due to competing comorbidities.^20^ The key components of CGA include assessment of physical function, cognition, comorbidities, nutrition, polypharmacy, and psychologic and social evaluation. The types and number of medications can reflect comorbidities and current health status. As medication review is one of the core components of CGA, it may be a potential surrogate to indicate which patients should go on to receive a full CGA.

The aims of this present study are to (1) analyze the prevalence of medication use in a cohort of older women with early-stage operable primary breast cancer; (2) assess the relationship of medication use with primary treatment decision that was made; and (3) assess the relationship of medication use with functional status as measured by a cancer-specific CGA.^21^ In light of the prevalence of cardiovascular drugs shown in our results, we have also assessed the relationship between cardiovascular-related disease and primary treatment decision, and the relationship between cardiovascular-related disease and functional status.

## Methods

This study is part of an ongoing prospective study in older women with primary operable breast cancer from 3 UK centers. The study design has been described in detail previously^22^ and is summarized below.

### Participant Recruitment

Women attending the breast cancer clinic who were newly diagnosed with breast cancer and met the criteria below were invited to participate in the study. Treatment decisions were made after consultation with the medical team and not guided by any aspect of the study.

Inclusion criteria included age ≥70 years; new diagnosis of clinically early operable primary breast cancer. Patients who had received prior treatment for breast cancer, who had evidence of metastatic disease, were not able to consent were excluded. Hormone receptor status or further clinicopathological features were not collected as part of CGA. Anecdotally, this is a cohort of older women with primary breast cancer, so the majority will be hormone receptor positive, human epidermal growth factor receptor 2 (HER2) negative.

### Data Collection

For the patients who consented to participate in the study, a cancer-specific validated CGA^21^ was administered within 6 weeks of diagnosis (Supplementary Material). On completion of the CGA, we extracted the key domains that are of particular interest to this study (See Appendix A in Supplementary Material). These are physical function, comorbidities, and polypharmacy. Thus we have selected the following CGA items: cancer treatment received; ability of patient to independently take their medications; performance rating by both patients and clinicians; number and impact of comorbidity; timed up and go (TUG) test; number of falls in last 6 months; and ability to perform activities of daily living (ADL) and instrumental activities of daily living (IADL), in order to compare with patients’ medications. Medications, as captured in the patent questionnaires of CGA, were sorted by a clinical pharmacist according to the International Non-proprietary Names and Anatomical Therapeutic Chemical codes recommended by the World Health Organization.^23^ The number of medications was recorded, where polypharmacy and hyperpolypharmacy were defined as 5 or more and 10 or more daily medicines, respectively. Standard scoring guidelines were used to score each component of the CGA.^21^

### Additional Analysis

The most prevalent classes of medication prescribed in this cohort were related to cardiovascular disease; therefore, an additional analysis was performed to determine if patients with cardiovascular-related diseases (as measured by CGA) compared to non-cardiovascular diseases was a significant factor. Cardiovascular-related diseases included high blood pressure, heart disease, circulation problems, and stroke, whilst non-cardiovascular-related diseases included cancers (other than breast cancer), arthritis, glaucoma, emphysema, diabetes, stomach or intestinal disorders, osteoporosis, and chronic liver or kidney.

### Statistical Analysis

All data extracted from the CGA were coded, de-identified, and processed with the Statistical Package for the Social Sciences version 28 (SPSS, Chicago, IL).

Descriptive data were treated as categorical variables with the exception of number of medications; the number and impact of comorbidities which were treated as continuous variables. Chi-squared test was used to compare 2 categorical variables, Fisher’s exact test was used to compare 2 categorical variables when the number of cases in 1 variable was <5, and Kruskal–Wallis was used to compare a continuous variable with 2 or more categorical variables. Spearman’s correlation was used to compare 2 continuous variables.

For all tests, a significant difference was considered if *P* < .05. Multivariate analysis was performed using multiple regression to determine which factors of CGA were independently significant for predicting a greater number of daily medications.

## Results

### Description of the CGA Results from Nottingham Centers

Analysis of the full CGA has not yet been performed or published but here are the overall results from a pilot study of *N* = 47 patients.^22^ Increasing age (≥80 years) (*P* = .001), greater (≥4) comorbidity (*P* = .022), greater number (≥4) of daily medications (*P* = .002), and slower (≥19 s) TUG (*P* = .016) score were significantly related to nonsurgical treatment at 6 weeks after diagnosis.^22^ At 6-month post-diagnosis, age was the only factor significantly associated with patients having nonoperative treatment (*P* < .001).^22^ Patients over 80 years were more likely to receive PET than surgery.^22^

### Description of Cohort

Data from a total of 139 patients with an average age of 77 years (range 68–93) were extracted. Of the 139 patients, 116 (83%) had primary surgery and 23 (17%) had PET.

### Prevalence of Medication Use

Among the 120 patients with complete drug histories and stated number of daily medications, 57 (48%) had polypharmacy and 11 (9%) had hyperpolypharmacy (Table 1). Patients took an average of 4 daily medications (range 0–15). Table 2 shows the 9 most prevalent classes of medications. The majority of these (66.7%) are used to treat cardiovascular conditions (marked with an *).

**Table 1.** Prevalence of medication use.

	Patients with <5 daily medications	Patients with polypharmacy (≥5 daily medications)	Patients with hyperpolypharmacy (10 or more daily medications)
Number of patients	63	57	11
Percentage	52%	48%	9%

**Table 2.** Most prevalent classes of medications amongst the study cohort.

ATC	Drug	Total cohort (*n* = 120)	Percentage
C10	Lipid modifying agents^a^	46	38.3%
C09	Agents acting on the renin–angiotensin system^a^	41	34.2%
C03	Diuretics^a^	38	31.7%
N02	Analgesics	31	25.8%
A02	Drugs for acid-related disorders	30	25.0%
B01	Antithrombotic agents^a^	29	24.2%
C08	Calcium channel blockers^a^	25	20.8%
C07	Beta-blocking agents^a^	19	15.8%
	Over-the-counter supplements^b^	16	13.3%

^a^Medications used to treat cardiovascular conditions.

^b^Over-the-counter supplements and miscellaneous items (topicals, nasal spray) are not classified under ATC.

Of the139 patients, 110 (79%) reported they could take their medications without help and 28 (20%) needed some help. The number of daily medications prescribed did not affect the ability of patients to take their medications (*P* = .218). Requiring help with taking medications was associated with receipt of PET (compared to surgery) (*P* = .002) (Table 3).

**Table 3.** Association between patient’s ability to take medications and PET.

Ability to take medications	Surgery (%)	PET (%)	Total	*P* value
With some help	18 (64%)	10 (36%)	28	.002
Without help	97(88%)	13 (12%)	110	

### Association Between Number of Medications and Early-Stage Operable Primary Breast Cancer Treatment

Among the 139 patients, those taking a greater number of daily medications (as a continuous variable) were more likely to receive nonoperative treatment (*P* = .036) (Table 4). There was no association between polypharmacy (*P* = .233) or hyperpolypharmacy (*P* = .879) and primary treatment undertaken (Tables 5 and 6).

**Table 4.** Association between number of daily medications and type of treatment.

	Number of patients	Mean of daily medications	Significance
Surgery	116	4.07 (SD = 3.248)	*P* = .036 [CI −3.06, −0.106]
Nonoperative treatment	23	5.65 (SD = 3.393)	

**Table 5.** Association between polypharmacy and type of treatment.

	Polypharmacy	Non-polypharmacy	Pearson Chi-Square
Surgery	45	71	*P* = .233
Nonoperative treatment	12	11	

**Table 6.** Association between hyperpolypharmacy and type of treatment.

	Hyperpolypharmacy	Non-polypharmacy	Pearson Chi-Square
Surgery	9	107	*P* = .879
Nonoperative treatment	2	21	

### Association Between Medication Use and Functional Status

Table 7 shows patients with a higher number of daily medications were associated with greater comorbidities and reduced performance status.

**Table 7.** Associations between number of medications and comorbidities and physical status.

	Outcome measures	Results
Number of daily medications	Comorbidities, PS, ADLs, and IADLs	Comorbidities (*P* < .001); PS rated by physician (*P* = .009); PS rated by patients (*P* = .002); ADLs (*P* = .009); IADLs (*P* < .001)
Polypharmacy	Comorbidities, PS, and IADLs	Comorbidity (*P* < .001); PS rated by the physician (*P* = .009); PS rated by patients (*P* = .019); IADLs (*P* = .008)
Hyperpolypharmacy	Comorbidities and PS	Comorbidity (*P* = .006); PS rated by the physician ( *P* = .009); PS rated by patients (*P* = .019)

A greater number of prescribed daily medications were associated with greater number and impact of comorbidities, reduced performance status rated by the physician and the patient, and reduced ability to perform ADLs and IADLs.

### Additional Analysis Relating to Cardiovascular Disease

Out of 139 Participants, 42 patients (30%) had no cardiovascular-related diseases whilst 97 patients (70%) had cardiovascular-related problems. Patients with cardiovascular-related diseases compared to non-cardiovascular diseases were more likely to take a greater number of daily medications (*P* < .001), have greater comorbidity and impact of illness (*P* < .001), have reduced ability to perform ADLs (*P* = .005) and IADLs (*P* = .001), and reduced ability to take medications independently (*P* = .017).

### Multivariate Analysis Results

The following variables were taken forward into multivariate analysis (as they were significant on the univariate analysis): greater number and impact of comorbidities; physician-rated performance status; patient-rated performance status; ADLs; iADLs; and greater likelihood of receiving PET as opposed to surgery (Table 8).

**Table 8.** Multivariate analysis comparing number of medications and factors of CGA and treatment.

Factor	*P* value
Item of CGA	
Comorbidity	.00
Physician-rated performance	.388
Patient-rated	.799
ADL	.009
IADL	.002
Treatment	.23

Note: ADL, IADL, and comorbidity were independently significantly associated with number of medications.

## Discussion

Polypharmacy was highly prevalent in this cohort of older women with breast cancer. Greater comorbidity was associated with reduced functional status and receipt of nonoperative treatment (compared to surgical treatment) and correlated with increasing number of daily medications.

### Description of Cohort

In this prospective study, polypharmacy and hyperpolypharmacy were present in 48% and 9% of our participants, respectively. Depending on the definition of polypharmacy and population studied in different settings, the prevalence of polypharmacy varies greatly, ranging from less than 10% to more than 90%.^24^ Using the same definition of taking five or more daily medications, 57% of patients ≥70 years with gastrointestinal, lung, breast, prostate, or hematological cancer who attended oncology outpatient clinic presented with polypharmacy.^8^ In a study of 500 community-dwelling older patients with cancer including solid tumors and hematology-related cancer, the prevalence of polypharmacy and hyperpolypharmacy were 41% and 43%, respectively.^25^ To the best of our knowledge, there is no study on the prevalence of polypharmacy in older women with early-stage operable primary breast cancer. The findings in this present study are in keeping with the literature above.

### Prevalence of Medication Use

Cardiovascular-related drugs are the most prevalent class of medicines in this cohort, which is similar to Topaloğlu’s study.^26^ This should not be a surprise, as treatment guidelines that are developed for the management of a single condition require a combination of drugs to achieve prognostic and therapeutic benefits. However, the majority of these medications are associated with admission due to adverse drug effects (ADEs).^11^ Examples include ACE inhibitors and diuretics, which are common culprits for acute kidney injury; beta-blockers and calcium channel blockers can cause postural hypotension; anticoagulants, and antiplatelets can lead to bleeding; statins can cause muscle weakness. These presentations can manifest as common geriatric syndromes such as falls and can affect a patient’s functional status, which could be of concern in older patients with cancer. In the additional analysis, cardiovascular diseases were associated with greater comorbidities and reduced physical status. This could be due to the disease itself as well as polypharmacy.

In this study, we did not seek to individually identify inappropriate polypharmacy according to existing criteria such as Beers or STOPP/START. However, this may be of potential value as polypharmacy and potentially inappropriate medication (PIM) are common in older patients with cancer.^27^ Karuturi and colleagues conducted a study investigating PIM in older patients with breast and colorectal cancer.^28^ PIM was identified in 22%–27% of the breast cohort and correlated with polypharmacy and other adverse outcomes such as hospitalization and increased mortality.^28^ In Mohamed’s systematic review, polypharmacy was associated with chemotherapy toxicities, falls, and functional decline, and PIM was associated with adverse clinical outcomes.^27^ Having a pharmacist as part of a multidisciplinary team with the use of medication screening tools may be beneficial not only for medication review^25,29,30^ but also to identify the more frail women who should receive a full CGA.

### Association Between Number of Medications and Early-Stage Operable Primary Breast Cancer Treatment

The present study found that patients with a greater number of daily medications were more likely to receive PET. As mentioned in our introduction, various thresholds for polypharmacy and hyperpolypharmacy have been confirmed as predictors for hospital admission, adverse outcomes, falls, medication compliance, and mortality.^31–33^ In a study of polypharmacy in community-dwelling older men, the use of 5 or more medications was shown to be a reasonable cutoff and was associated with falls, disability, and frailty.^34^ Hyperpolypharmacy (using a definition of consumption of 10 or more daily drugs) has been consistently shown to be a predictor of ADEs in the older population.^6,33,35,36^ A cohort analysis involving over 180 000 patients demonstrated that the effect of increasing the number of medications to hospitalization was diminished in patients with multiple conditions.^37,38^ Patients with single disease who were taking between 4 and 6 medications were more likely to have unplanned admission compared to those on 1–3 medications. However, amongst the patients who had 6 or more comorbidities, those who took between 4 and 6 regular medications did not have a significantly higher incidence of admission.

Therefore, there is no “one size fits all” definition that is suitable in practice. Taking a greater number of medications does not necessarily mean that patients were not fit enough for surgery. We should consider the clinical context and stratify patients at high risk for ADE when interpreting polypharmacy.^6^

As the decision for treatment is not influenced by the outcomes of the CGA in the original study, other factors such as patient choice or frailty could influence the treatment decision. Patients who have polypharmacy might be reluctant to have surgery due to a number of health conditions. It is beyond the scope of this study to address the former; however, frailty plays an important role in predicting surgical outcomes. We recognize that differentiation by tumor type and other treatment approaches have not been included in this study and this may also impact treatment decision-making.

### Association Between Medication Use and Functional Status

The present study found that the greater number of daily medications was associated with greater number and impact of comorbidities, impaired physical function including reduced performance status and reduced ability to perform ADLs and IADLs, in older patients with breast cancer. On multivariate analysis, the ability to perform ADLs and IADLs and greater number and impact of comorbidities were independently significant variables.

This agrees with previous studies which showed that patients with polypharmacy were likely to have a greater number of comorbidities, more PIMs related to adverse outcomes, and poor physical functional impairment in older adults with cancer.^1^, Mohamed et al.^27^

A recent study investigating the association between polypharmacy and mortality in patients with colorectal cancer^39^ found that, in a cohort of 3239 patients, 54.7% of them had polypharmacy (5 or more daily medications).^39^ For those taking 8 or more medications, they were more likely to have reduced 5-year overall survival and colorectal cancer–specific survival.^39^ The study also observed that poorer functional status was associated with polypharmacy.^39^ In Prithviraj’s study, it also found that polypharmacy was associated with the Eastern Cooperative of Oncology Group performance status score.^40^

An interesting finding was a lack of association between number of medications and TUG test. Ozkok and colleagues also found similar findings when comparing polypharmacy with physical performance status in older adults.^41^ Although TUG test is an objective assessment, there is a lack of studies to show its association with polypharmacy.^41^ In fact, it is a single test that is commonly used to screen patients with a risk of falls.^42^ Reduced physical function can be multifactorial, age related, or disease related. Appropriate polypharmacy may actually improve physical status.

### Additional Analysis Relating to Cardiovascular Diseases

Despite the high prevalence of cardiovascular drugs in this cohort, patients with cardiovascular disease were not any more likely to receive nonoperative treatment. There is a lack of evidence showing the impact of cardiovascular status on breast cancer treatment decision.^43^ However, in light of potential cardiotoxicity induced by radiation or some chemotherapy such as anthracycline and trastuzumab, cardiovascular assessment is recommended prior to breast cancer treatment.^44^

In our study, patients with cardiovascular diseases are more likely to have polypharmacy, which can easily be explained by a combination of drugs commonly seen in cardiovascular disease or heart failure or stroke. However, they did not necessarily have reduced performance status. This echoes our point that polypharmacy can be appropriate and beneficial to patients. The risk of polypharmacy should be judged on an individual basis.

### Limitations of the Present Study

One of the limitations of this study is that medications were captured by patients only; however, this is part of the validated CGA tool. We were unable to validate the accuracy of the data or comment on patients’ compliance. In future, such information can be verified using medical notes or electronic records. This will also help us to determine the appropriateness of polypharmacy for an individual in relation to their functional status. This was a cross-sectional study, and therefore, only an association between polypharmacy and outcomes could be concluded. The number of our cohort is low; however, to the best of our knowledge, this is still the largest study of its kind investigating this subject. In general, older people are underrepresented in research. Our study is ongoing. The definition of polypharmacy remains a challenge; however, we have adapted the most common definition in the literature. Although our work did not include a correlation with tumor biology, the study focuses on treatment decisions from the perspective of geriatric domains, whilst we have a parallel study going on focusing on tumor biology.

## Conclusion

In summary, patients with a greater number of medications are more likely to have multiple comorbidities and are more likely to have functional limitations. Although there may be no association between polypharmacy and other CGA components, we are leading on to show that the number of medications could be used as a screening tool for who should go on to receive full CGA.

Nevertheless, we should be mindful that appropriate polypharmacy can be beneficial to patients. Treatment decision-making in older women with breast cancer should be made following consultation with the multidisciplinary team, application of CGA where possible and discussion of the individual’s preferences.^45–57^

## Supplementary Material

oyac278_suppl_Supplementary_Material

## Data Availability

The data underlying this article cannot be shared publicly due to the privacy of individuals that participated in the study. The data will be shared on reasonable request to the corresponding author.
