# Supplementary material for: The Association Between Medication Use in Older Women with Early-Stage Operable Primary Breast Cancer and Decision Regarding Primary Treatment
Source: Oncologist. 2023 Jan 30;28(3):e128–35. doi: 10.1093/oncolo/oyac278 (PMC10020815; doi:10.1093/oncolo/oyac278)
Supplement: oyac278_suppl_Supplementary_Material [file oyac278_suppl_supplementary_material.docx]

**Components to be completed by the participant:**

| **Question number** | **Question Title** | **Measure** | **Scoring and analysis** |
| --- | --- | --- | --- |
| 1 | Your Background | Education, marital status, who lives at home, employment status, age, ethnicity | - Categorical variables - Age – single continuous variable |
| 2 | Your daily activities | Activities of daily living (ADL) | Total of 7 activities scored from 0 – 2 regarding ability to perform (maximum score 14, indicating best level of activity) |
| 3 | Daily activities | Instrumental activities of daily living (IADL) | Total of 10 activities scored from 0 – 2 regarding lack of limitation (maximum score 30, indicating least limitation) |
| 4 | Performance Rating (PR) Scale | Karnofsky self-reported performance rating scale | Categorical scale from 1 – 8 defining best PR |
| 5 | Falls | Number of falls in last 6 months | Single continuous variable |
| 6 | Your medications | Named medication, dose and frequency | Total number of named medications, regardless of dose or frequency |
| 7 | Your Health | Physical Health Section (subset of the Older American Resources and Services (OARS)) | Total of 17 comorbidities scored 1 if present, plus rating from 0 – 2 depending on interference with daily activities (total score for each comorbidity therefore 3, maximum score 51, indicating most comorbidity) |
| 8 | Your Nutritional status | Percentage unintentional weight loss in last 6 months | Single continuous variable |
| 9 | Your Mood | Hospital Anxiety and Depression Scale (HADS) | Total of 17 symptoms related to mood scored on scale from 1 – 6 depending on frequency (maximum score 102, indicating better mood) |
| 10 | Your Social Activities | Medical Outcomes Study (MOS) Social Activity Limitations Measure | Total of 4 questions rated on scale from 1-5 depending on frequency/ severity (maximum score 20, indicating most socially active) |
| 11 | Your Social Support | MOS Social Support Survey Seeman and Berkman Social Ties | 12 questions rated on scale from 1 – 5 depending on frequency (maximum score 60, indicating most socially supported) |
| 12 | Feedback | Ability to understand questions / length of questionnaire / any questions left out / upsetting | - Categorical variable - Free text |

**Components to be completed by the researcher:**

| **Question number** | **Question Title** | **Measure** | **Scoring and analysis** |
| --- | --- | --- | --- |
| 1 | Medical characteristics | Cancer type/stage/ chemotherapy regimen | Categorical variables |
| 2 | Karnofsky performance scale (KPS) | Karnofsky physician-rated performance rating scale | Categorical scale from 0 – 100 defining best performance |
| 3 | Timed ‘up and go’ (TUG) | Timed ‘up and go’ (TUG) | Single continuous variable |
| 4 | Cognition | Blessed Orientation-Memory-Concentration test (BOMC) | Single continuous variable |
| 5 | Nutrition | Body mass index (BMI) | Single continuous variable |
| 6-9 | Feedback | Time to complete/ assistance to complete | - Time - single continuous variable - Assistance – single categorical variable |
